# TPGS Analog-Mediated Intracellular ROS-Amplifying Strategy Potentiates the In Vitro Anticancer Activity of a Dual-Thioketal-Linked Polymeric Drug Conjugate in A549 Lung Cancer Cells

**DOI:** 10.3390/pharmaceutics18070886

**Published:** 2026-07-20

**Authors:** Hyun-Chul Kim, Kyeong-Min Lee, Yeo Jin Hwang, Jonghun Lee, Hwa Seung Han

**Affiliations:** 1 Division of Biomedical Technology, Daegu Gyeongbuk Institute of Science and Technology (DGIST), Daegu 42988, Republic of Korea; kimhc@dgist.ac.kr (H.-C.K.); leekm1009@dgist.ac.kr (K.-M.L.); 2 Division of AI, Big Data and Block Chain, Daegu Gyeongbuk Institute of Science and Technology (DGIST), Daegu 42988, Republic of Korea; yjhwang@dgist.ac.kr; 3 Division of Automotive Technology and the Department of Interdisciplinary Engineering, Daegu Gyeongbuk Institute of Science and Technology (DGIST), Daegu 42988, Republic of Korea

**Keywords:** reactive oxygen species, thioketal, camptothecin, polymeric drug conjugate, TPGS, lung cancer

## Abstract

**Background/Objectives**: Reactive oxygen species (ROS)-responsive polymeric drug conjugates (PDCs) can enable oxidative stress-triggered drug release, but their activation may be limited by heterogeneous or insufficient intracellular ROS. Herein, we synthesized a dual-thioketal-linked PDC bearing two ROS-cleavable thioketal (TK) units in series and combined it with D-α-Tocopheryl polyethylene glycol succinate analog (TPGSa) as a soluble ROS-modulating co-treatment. **Methods**: PDC was synthesized through stepwise construction of the TK linker and subsequent carbonate coupling with camptothecin (CPT). TPGSa was prepared by esterifying mPEG with tocopheryl succinate. PDC nanoassembly formation, colloidal stability, peroxide-induced structural changes, thiol generation, and CPT release behaviors were evaluated under oxidative conditions. Cytotoxicity was examined in A549 and BEAS-2B cells with intracellular ROS- and CPT-associated fluorescence. **Results**: PDC formed spherical nanoassemblies with a hydrodynamic diameter of 98.6 ± 2.6 nm and a zeta potential of −13.3 ± 1.2 mV. The PDC remained colloidally dispersed in 10% FBS-containing PBS and after lyophilized storage. Peroxide exposure produced concentration-dependent thiol generation, molecular size change, and CPT release. The PDC + TPGSa reduced A549 viability more than PDC alone, produced the most pronounced dead-cell staining, and yielded the highest intracellular ROS and CPT fluorescence signals. In contrast, BEAS-2B viability remained substantially higher under matched conditions. **Conclusions**: These findings support an A549-focused in vitro proof of concept in which TPGSa-associated redox perturbation is paired with a dual TK PDC to enhance CPT-associated cytotoxicity.

## 1. Introduction

Camptothecin (CPT) is a potent topoisomerase I inhibitor and a clinically validated scaffold for cancer chemotherapy, but its direct therapeutic application is limited by poor aqueous solubility and pH-dependent conversion of the active lactone to the inactive carboxylate form under physiological conditions [1,2]. Polymeric drug conjugate (PDC) can improve the aqueous handling of CPT, reduce premature drug leakage, and provide a chemical framework for stimulus-responsive release [3,4]. Conjugation through the 20-hydroxyl group of CPT is widely used to mask the parent drug behind a cleavable linkage, which makes the linker a central determinant for conjugate stability and drug liberation [5].

Reactive oxygen species (ROS)-responsive linkers are attractive because many cancer cells exhibit elevated oxidative stress relative to non-cancerous cells, providing a redox difference that has been explored for anticancer drug release [6,7,8,9,10,11,12,13]. Among ROS-cleavable motifs, thioketal (TK) linkers have been widely used owing to their ROS-mediated oxidative cleavage under ROS-rich conditions while remaining comparatively stable under non-oxidative physiological conditions [14,15,16]. Existing TK-based delivery designs generally fall into two structural categories: single-TK polymer–drug conjugates, which place one oxidative cleavage site at the polymer–drug junction [17,18], and multi-TK polymer backbones, in which repeated TK cleavage destabilizes or degrades the carrier framework [14,19]. These arrangements leave a distinct linker-level design opportunity: positioning multiple TK units in series at the polymer–drug linker to increase the ROS-labile motifs at the drug release junction.

A key limitation of ROS-responsive PDCs is that their activation depends on the level, localization, and lifetime of intracellular ROS. Endogenous antioxidant systems can buffer ROS, and ROS-rich cancer cells may still provide insufficient or transient oxidative input for timely linker cleavage [7,9,20]. In particular, endogenous ROS may be insufficient in some cancer cell types, particularly those with modest basal oxidative stress, to sustain timely and efficient cleavage of ROS-responsive linkers. This trigger-limited behavior has motivated pairing an ROS-responsive PDC with a separately administered redox-modulating component.

D-α-tocopheryl polyethylene glycol 1000 succinate (TPGS) is an amphiphilic vitamin E derivative widely used as a solubilizer, formulation stabilizer, and P-glycoprotein modulator [21,22]. Beyond these formulation roles, TPGS and related tocopheryl succinate-derived amphiphiles have been associated with mitochondrial perturbation, increased intracellular ROS signals, and the potentiation of anticancer drugs in selected models [7,23,24,25]. We therefore prepared mPEG2k-tocopheryl succinate (TPGSa), a PEG2000-based TPGS analog, for use as a co-administered redox-modulating component for the ROS-responsive PDC system. The TPGSa retains the tocopheryl succinate motif with TPGS-inspired mitochondrial perturbation and ROS modulation, while its mPEG2k segment provides hydrophilicity, supports aqueous solubility, and matches the PDC composition.

Based on this rationale, we hypothesized that TPGSa-associated redox perturbation would support oxidative activation of a dual TK PDC and thereby increase CPT-associated cytotoxicity in A549 cells. The linear PDC was designed to self-assemble into PEG-shielded CPT-containing nanoassemblies. Cleavage of the mPEG-proximal TK was proposed to weaken the PEG-shielded architecture, whereas cleavage of the CPT-proximal TK was proposed to generate a thiol-containing carbonate species capable of subsequent CPT liberation through a self-immolative process [26].

**Figure 1:**
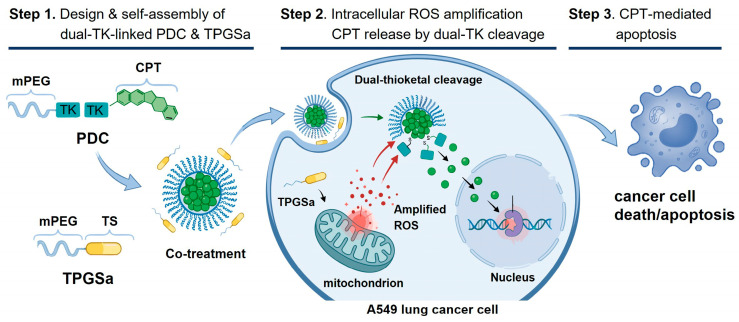
Schematic illustration of the proposed co-treatment strategy combining the polymeric drug conjugate (PDC) and the mPEG2k–tocopheryl succinate TPGS analog (TPGSa) in A549 lung cancer cells. Step 1: linear PDC self-assembles into PEG-shielded, CPT-containing nanoassemblies in aqueous media, whereas TPGSa is administered as a separate soluble co-treatment. Step 2: TPGSa-associated redox perturbation increases intracellular ROS-associated signals and supports oxidative cleavage of the TK-containing linker region, nanoassembly destabilization, and CPT liberation. Step 3: liberated CPT induces A549 cell death.

Herein, we report the synthesis, physicochemical characterization, peroxide responsiveness, and in vitro biological evaluation of the PDC in combination with TPGSa. A549 lung adenocarcinoma cells served as the cancer model and BEAS-2B bronchial epithelial cells as a non-cancerous comparison model. The study is positioned as an A549-focused in vitro proof of concept rather than as evidence of dual-TK superiority or in vivo therapeutic performance. The proposed overall design and schematic illustration are summarized in Figure 1.

## 2. Materials and Methods

### 2.1. Materials

mPEG (Mw 2000 g/mol), 4-dimethylaminopyridine (DMAP), 1-(3-dimethylaminopropyl)-3-ethylcarbodiimide hydrochloride (EDC), 3-mercaptopropionic acid, triphosgene, and 2′,7′-dichlorofluorescein diacetate (DCFH-DA) were purchased from Sigma-Aldrich Corp. (St. Louis, MO, USA). CPT and α-tocopherol succinate were purchased from Tokyo Chemical Industry Co., Ltd. (Tokyo, Japan). Cell Counting Kit-8 (CCK-8) was obtained from Dongin LS (Seoul, Republic of Korea). Roswell Park Memorial Institute (RPMI) 1640 medium, fetal bovine serum (FBS), and penicillin–streptomycin solution were purchased from Welgene Inc. (Gyeongsan, Republic of Korea). The LIVE/DEAD Viability/Cytotoxicity Kit was obtained from Thermo Fisher Scientific (Waltham, MA, USA). A549 human lung adenocarcinoma cells and BEAS-2B human bronchial epithelial cells were obtained from the American Type Culture Collection (ATCC, Manassas, VA, USA). All solvents were of reagent grade and were used without further purification unless otherwise stated.

### 2.2. Methods

#### 2.2.1. Synthesis of PDC and TPGSa

The dual-thioketal polymeric drug conjugate mPEG-TK-TK-CPT (PDC) was synthesized via a stepwise route that places two thioketal (TK) motifs in series within the mPEG-CPT linker region. The sequence included: (i) preparation of diethyl thioketal (DET) and propanediol thioketal (PDT) as bifunctional TK building blocks; (ii) coupling of mPEG to DET to obtain mPEG-TK (Compound 1); (iii) extension with PDT to obtain mPEG-TK-TK (Compound 2); and (iv) carbonate-mediated conjugation of Compound 2 with CPT through the 20-OH group of CPT to afford PDC. The mPEG-tocopheryl succinate conjugate (TPGS analog; hereafter TPGSa) was synthesized separately by EDC/DMAP-mediated esterification of mPEG with α-tocopherol succinate.

#### 2.2.2. Synthesis of Thioketal Linkers

Diethyl thioketal (DET; 3,3′-(propane-2,2-diylbis(sulfanediyl))dipropanoic acid) was synthesized according to a previously reported procedure [15]. Briefly, acetone (3 mL) was mixed with 3-mercaptopropionic acid (9 mL) and refluxed under stirring for 72 h. The reaction mixture was poured into ice water, and the resulting white crystals were filtered, washed three times with ice-cold water, and lyophilized to afford DET as a white solid. ^1^H-NMR (400 MHz, CDCl_3_): δ 1.62 (s, 6H, -C(CH_3_)_2_-), 2.70 (t, 4H, -S-CH_2_-), 2.92 (t, 2H, -CH_2_-COOH).

Propanediol thioketal (PDT; 3,3′-(propane-2,2-diylbis(sulfanediyl))bis(propan-1-ol)) was prepared by reducing DET with lithium aluminum hydride (LiAlH_4_). DET (3 g) was dissolved in anhydrous tetrahydrofuran (THF, 150 mL), and LiAlH_4_ (6 g) was added slowly in an ice bath. The mixture was refluxed at 60 °C for 4 h under a nitrogen atmosphere. After removal of THF under reduced pressure, aqueous NaOH (15%, 200 mL) was added to quench excess LiAlH_4_. The mixture was filtered, and the filtrate was extracted with ethyl acetate, yielding PDT as a colorless oil. ^1^H-NMR (400 MHz, CDCl_3_): δ 1.62 (s, 6H, -C(CH_3_)_2_-), 1.86 (t, 2H, -CH_2_-CH_2_-OH), 2.76 (t, 2H, -S-CH_2_-), 3.76 (t, 2H, -CH_2_-OH).

#### 2.2.3. Synthesis of Compound **1**

DET (2.52 g, 10.0 mmol), EDC (0.32 g, 1.68 mmol), and DMAP (0.02 g, 0.16 mmol) were dissolved in anhydrous dichloromethane (DCM, 100 mL). mPEG (2.0 g, 1.0 mmol) was then added slowly, and the reaction mixture was stirred at room temperature for 24 h under a nitrogen atmosphere. The organic layer was washed with distilled water, separated, dried over anhydrous MgSO_4_, and concentrated under reduced pressure. The concentrate was precipitated into excess cold diethyl ether, collected, and dried under vacuum to afford mPEG-TK as a white powder (1.80 g, approximately 80% yield). ^1^H-NMR (400 MHz, CDCl_3_): δ 1.62 (s, 6H, -C(CH_3_)_2_-), 2.69 (t, 4H, -S-CH_2_-), 2.91 (t, 2H, -CH_2_-COOH), 3.40 (s, 3H, -OCH_3_ of mPEG), 3.55–3.84 (m, -OCH_2_CH_2_O- backbone), 4.28 (t, 2H, mPEG-OCH_2_CH_2_-OC(O)-).

#### 2.2.4. Synthesis of Compound **2**

Compound **1** (1.50 g, 0.67 mmol), PDT (1.50 g, 6.7 mmol), EDC (0.19 g, 1.0 mmol), and DMAP (0.03 g, 0.24 mmol) were dissolved in anhydrous DCM (50 mL) and stirred at room temperature for 24 h under a nitrogen atmosphere. The mixture was washed with distilled water, separated, dried over anhydrous MgSO_4_, and concentrated under reduced pressure. The concentrate was precipitated into excess cold diethyl ether, collected, and dried under vacuum to afford Compound 2 as a white powder (1.38 g, approximately 85% yield). ^1^H-NMR (400 MHz, CDCl_3_): δ 1.62 (s, 6H), 1.86 (t, 2H), 2.75 (t, 2H), 2.88 (t, 4H), 2.91 (t, 2H), 3.40 (s, 3H), 3.55–3.84 (m), 4.20 (s, 2H), 4.28 (t, 4H).

#### 2.2.5. Synthesis of PDC

CPT (0.20 g, 0.57 mmol) and DMAP (0.21 g, 1.72 mmol) were suspended in anhydrous DCM (20 mL) under a nitrogen atmosphere. Triphosgene (0.06 g, 0.20 mmol) was added, and the mixture was stirred at room temperature for 2 h to activate the 20-OH group of CPT as a chloroformate intermediate. A solution of Compound 2 (1.0 g) in anhydrous THF (10 mL) was then added dropwise, and the reaction was stirred for an additional 24 h. The mixture was filtered, and the solvent was removed under reduced pressure. The residue was redissolved in DCM, washed twice with 1.0 M aqueous HCl and twice with saturated brine, and the organic phase was dried over anhydrous MgSO_4_. After concentration under reduced pressure, the concentrate was precipitated into excess cold diethyl ether to afford PDC as a pale-yellow powder (0.86 g). ^1^H-NMR (400 MHz, CDCl_3_): CPT aromatic signals at 7.4–8.4 ppm, TK methyl resonance at 1.62 ppm, mPEG backbone resonance at approximately 3.6 ppm, and carbonate-adjacent methylene signals around 4.0–4.5 ppm.

A TK-free carbonate-linked mPEG2k-CPT conjugate was synthesized as a linker-insensitive control (LIC). CPT (0.25 g, 0.72 mmol) and DMAP (0.27 g, 2.16 mmol) were suspended in anhydrous DCM (10 mL) under nitrogen. Triphosgene (0.07 g, 0.23 mmol) was added, and the mixture was stirred for 2 h. A solution of mPEG2k-OH (1.40 g, approximately 0.70 mmol) in anhydrous THF (20 mL) was added dropwise, and the reaction was stirred for 24 h at room temperature. After filtration and solvent removal, the residue was dissolved in DCM, washed twice with 1.0 M HCl and twice with brine, dried over anhydrous MgSO4, concentrated, and precipitated into excess cold diethyl ether. LIC was obtained as a yellowish powder (1.28 g). ^1^H-NMR (400 MHz, CDCl_3_): CPT aromatic signals at 7.4–8.4 ppm, mPEG backbone resonance at approximately 3.6 ppm, methoxy signal at 3.4 ppm, carbonate-adjacent methylene signals around 4.0–4.5 ppm, and the CPT ethyl methyl signal near 1.0 ppm.

#### 2.2.6. Synthesis of TPGSa

TPGSa was synthesized by carbodiimide-mediated esterification between mPEG and α-tocopherol succinate. mPEG (2.0 g, 1.0 mmol), α-tocopherol succinate (0.64 g, 1.2 mmol), EDC (0.23 g, 1.2 mmol), and DMAP (0.15 g, 1.2 mmol) were dissolved in anhydrous DCM (100 mL) and stirred at room temperature for 24 h under a nitrogen atmosphere. The mixture was washed twice with distilled water, and the organic layer was separated, dried over anhydrous MgSO_4_, and concentrated under reduced pressure. The concentrate was precipitated into excess cold diethyl ether and dried under vacuum to afford mPEG2k-TS as a white powder (1.65 g, approximately 80% yield). ^1^H-NMR (400 MHz, CDCl_3_): chromanol-ring methyl signals around 1.8–2.1 ppm, phytyl-tail signals around 0.8–1.3 ppm, succinate CH_2_ signals at 2.5–2.7 ppm, and mPEG backbone resonance at approximately 3.6 ppm.

### 2.3. Physicochemical Characterization of PDC

^1^H-NMR spectra of all synthesized compounds were acquired on a Bruker AVANCE III 400 MHz NMR spectrometer at 25 °C using CDCl_3_ as solvent and tetramethylsilane (TMS) as the internal reference. Samples (5–10 mg) were dissolved in 0.6 mL of CDCl_3_, and 16–32 scans were collected per spectrum. Chemical shifts (delta) are reported in parts per million (ppm) and coupling constants (J) are reported in hertz (Hz).

The number-average molecular weight (Mn), weight-average molecular weight (Mw), and dispersity (Đ = Mw/Mn) of Compound **1**, Compound **2**, and PDC were determined by gel permeation chromatography (GPC) on a Waters 1515 system equipped with a Waters 2414 refractive index detector. Separation was performed on a Waters Styragel HR column at 35 °C using THF as the mobile phase at a flow rate of 1.0 mL/min. Calibration was performed using narrow-distribution poly(ethylene glycol) standards.

The hydrodynamic diameter and zeta potential of PDC were measured by dynamic light scattering (DLS) using a Malvern Zetasizer Nano ZS (Malvern Panalytical Ltd., Malvern, UK. PDC was dispersed in deionized water or PBS (1 mg/mL, pH 7.4), and samples were filtered through a 0.45 μm membrane before measurement. Each measurement was performed in triplicate at 25 °C using a backscattering angle of 173°.

PDC morphology was examined using transmission electron microscopy (TEM). PDC solution (1 mg/mL) was deposited onto a carbon-coated 200-mesh copper grid and air-dried at room temperature. The sample was negatively stained with 2% (*w*/*v*) phosphotungstic acid for 30 s, washed with deionized water, and air-dried. Images were acquired using a Hitachi HF-3300 transmission electron microscope (Hitachi High-Tech Corp., Tokyo, Japan) operated at an accelerating voltage of 300 kV.

Colloidal stability was evaluated by incubating PDC in PBS or PBS containing 10% (*v*/*v*) FBS at 37 °C. Hydrodynamic diameter was measured daily for 4 days using a Malvern Zetasizer Nano ZS. Storage stability was assessed using lyophilized PDC maintained at −20 °C. Samples were reconstituted at predetermined intervals over 28 days, and hydrodynamic diameter and zeta potential were measured using a Malvern Zetasizer Nano ZS.

### 2.4. ROS-Responsiveness and In Vitro CPT Release

#### 2.4.1. H_2_O_2_-Triggered Structural Change and CPT Release of PDC

To assess peroxide-triggered oxidative susceptibility of the TK-containing linker region, PDC was incubated in distilled water containing 1 mM H_2_O_2_ at 37 °C for 24 h. This condition was used as an accelerated peroxide condition for structural characterization and was not intended to reproduce intracellular ROS concentrations. GPC was used to assess changes in the apparent molecular weight and dispersity (Đ) of PDC before and after H_2_O_2_ treatment. Untreated PDC served as the non-oxidative control.

#### 2.4.2. DTNB/Ellman Thiol Detection Assay

H_2_O_2_-dependent generation of thiol species was evaluated using DTNB/Ellman analysis, an approach previously used to monitor ROS-triggered TK cleavage in nanomedicine systems [16]. PDC or LIC (1 mg/mL) was incubated in 0.1 M sodium phosphate buffer (pH 8.0) containing 1 mM EDTA and 0, 1 μM, 100 μM, or 1 mM H_2_O_2_. Aliquots were collected at 0, 0.5, 1, 2, 4, 6, 8, 12, and 24 h. Residual H_2_O_2_ was quenched with 100 U/mL catalase for 10 min at room temperature. Samples were then reacted with freshly prepared DTNB solution (4 mg/mL) for 15 min in the dark, and absorbance was measured at 412 nm. Thiol equivalents were calculated using an L-cysteine calibration curve. Five independently prepared reaction samples were analyzed per condition. LIC signals were interpreted as background-level because they remained below the manufacturer’s preferred quantitative range of 0.1–1.0 mM for all time points.

#### 2.4.3. In Vitro CPT Release

CPT release from PDC was evaluated using a dialysis method. PDC solution (1 mg/mL, 1 mL) was sealed in a dialysis membrane (molecular weight cut-off, MWCO 2 kDa) and immersed in 50 mL of PBS (pH 7.4) containing 0.1% (*w*/*v*) Tween 80 and at 0 μM, 1 μM, 100 μM, or 1 mM H_2_O_2_. Release studies were conducted at 37 °C with stirring at 100 rpm. At predetermined time intervals (0, 1, 2, 4, 8, 12, and 24 h), 3 mL of the external release medium was withdrawn and replaced with an equal volume of fresh medium maintained at 37 °C. The collected aliquots were lyophilized and redissolved in 50% acetonitrile/water. Released CPT was quantified by HPLC using an Agilent 1260 Infinity II system (Agilent Technologies, Santa Clara, CA, USA) equipped with a C18 reversed-phase column (4.6 × 250 mm, 5 μm). The mobile phase consisted of acetonitrile/water (45:55, *v*/*v*) at a flow rate of 1.0 mL/min, and UV detection was performed at 366 nm. CPT concentrations were calculated from a standard curve generated using free CPT. LIC was used as a TK-free chemical control.

### 2.5. Cell Culture

A549 and BEAS-2B cells were maintained in RPMI 1640 medium supplemented with 10% (*v*/*v*) FBS, 100 U/mL penicillin, and 100 μg/mL streptomycin. Cells were cultured in a humidified incubator at 37 °C under 5% CO_2_. The medium was refreshed every 2 days, and cells were sub-cultured at 70–80% confluence using 0.25% trypsin-EDTA. Cells between passages 3 and 15 were used for all experiments. Cells were routinely monitored for mycoplasma contamination, and experiments were performed with low-passage cells recovered from authenticated ATCC stocks.

### 2.6. In Vitro Anticancer Evaluation

#### 2.6.1. Cell Viability

Cell viability was determined using the Cell Counting Kit-8 (CCK-8) assay [27]. A549 cells (5 × 10^3^ cells/well) and BEAS-2B cells (1 × 10^4^ cells/well) were seeded in 96-well plates and allowed to attach overnight. Cells were treated with free CPT, PDC, TPGSa, or PDC plus TPGSa. CPT-containing treatments were tested at CPT-equivalent concentrations of 0.01, 0.1, 1, 10, and 20 μg/mL. The TPGSa was tested alone or co-administered with PDC at 0.1, 0.25, 0.5, 0.75, and 1.0 μg/mL, paired with the corresponding CPT-equivalent dose where applicable. PBS-treated cells served as the untreated control. After 24 h or 48 h of incubation, 10 μL of CCK-8 reagent was added to each well, and the plate was incubated for an additional 1 h at 37 °C. Absorbance at 450 nm was measured using a microplate reader (BioTek, Seoul, Korea). Five replicate wells were analyzed per condition (*n* = 5).

#### 2.6.2. Live/Dead Staining

A549 cells were seeded at 2 × 10^5^ cells/dish in 35 mm glass-bottom confocal dishes and cultured overnight. Cells were then treated with PBS (control), free CPT (20 μg/mL), PDC (20 μg/mL CPT-equivalent), TPGSa alone (1 μg/mL), or PDC plus TPGSa (20 μg/mL CPT-equivalent + 1 μg/mL TPGSa) for 15 h. After treatment, cells were stained using the LIVE/DEAD Viability/Cytotoxicity Kit (Thermo Fisher Scientific, Waltham, MA, USA) according to the manufacturer’s instructions. Briefly, the medium was aspirated and replaced with 2× staining working solution containing calcein-AM (live-cell stain, green fluorescence) and ethidium homodimer-1 (dead-cell stain, red fluorescence), and cells were incubated at 25 °C for 15 min. Fluorescence and bright-field images were acquired using an Olympus FV1200 laser-scanning confocal microscope (Tokyo, Japan) with FluoView Ver. 4.2a software at 200× magnification. Green and red fluorescence intensities were quantified using ImageJ software v1.8 software (National Institutes of Health, Bethesda, MD, USA) to estimate live- and dead-cell fractions, respectively.

### 2.7. Intracellular CPT Uptake and ROS Detection

#### 2.7.1. DCFH-DA Analysis

Intracellular ROS was assessed using the cell-permeant DCFH-DA probe, which is oxidized intracellularly by fluorescent 2′,7′-dichlorofluorescein (DCF) [28]. A549 cells were seeded in 35 mm glass-bottom confocal dishes (2 × 10^5^ cells/dish) and cultured overnight. Cells were treated with PBS (control), free CPT (20 μg/mL), PDC (20 μg/mL CPT-equivalent), TPGSa alone (1 μg/mL), or PDC plus TPGSa (20 μg/mL CPT-equivalent + 1 μg/mL TPGSa) for 24 h. After treatment, cells were washed twice with PBS and incubated with serum-free medium containing 20 μM DCFH-DA at 37 °C for 60 min. Cells were then fixed with 2% (*w*/*v*) cold paraformaldehyde for 15 min and washed twice with PBS. DCF fluorescence (λ_ex_/λ_em_ = 488/525 nm) was visualized using the same confocal microscope at 200× magnification. Integrated DCF fluorescence intensity per cell was quantified using ImageJ.

#### 2.7.2. CPT-Associated Fluorescence

Intracellular CPT was visualized using the intrinsic blue fluorescence of CPT (λ_ex_/λ_em_ = 352/402 nm). A549 cells were seeded in 35 mm glass-bottom confocal dishes (2 × 10^5^ cells/dish) and cultured overnight. The medium was then replaced with fresh medium containing PBS (control), free CPT (20 μg/mL), PDC (20 μg/mL CPT-equivalent), or PDC plus TPGSa (20 μg/mL CPT-equivalent + 1 μg/mL TPGSa), and cells were incubated for 4 h. After incubation, cells were washed twice with PBS, fixed with 2% (*w*/*v*) cold paraformaldehyde in PBS for 15 min, and rinsed twice with PBS. Intracellular CPT fluorescence was visualized using the same confocal microscope at 200× magnification. Integrated CPT fluorescence intensity per cell was quantified using ImageJ.

### 2.8. Statistical Analysis

All quantitative data are presented as mean ± standard deviation (SD) from n = 5 independently prepared samples. The results were scrutinized by two-way ANOVA for multiple group comparisons, and the results were analyzed statistically by one-way ANOVA for independent group comparisons using GraphPad Prism 10.3.1 software (GraphPad Software Inc., San Diego, CA, USA). Statistical significance is denoted as *p* < 0.05 (*), *p* < 0.01 (**), and *p* < 0.001 (***).

## 3. Results

### 3.1. Synthesis and Structural Characterization

**Figure 2:**
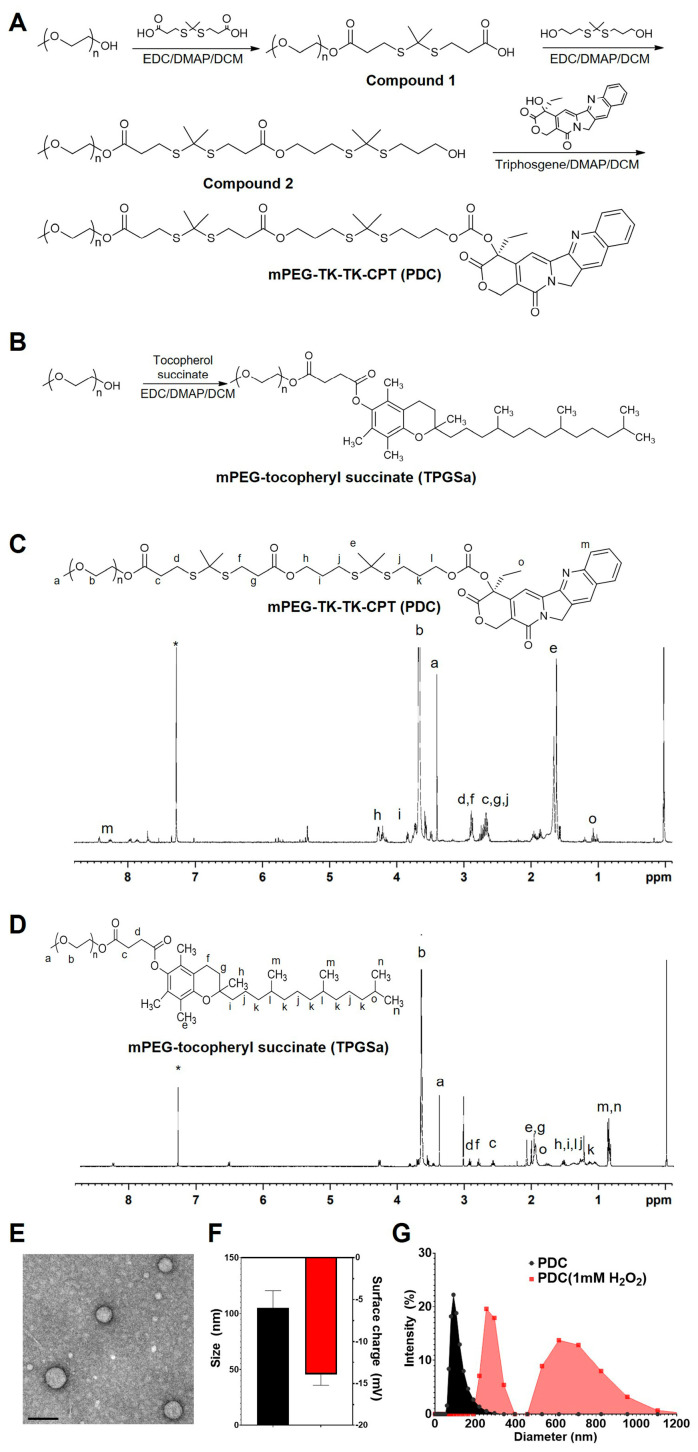
Synthesis and structural characterization of the PDC and TPGSa. (**A**) Synthetic route to PDC through Compound **1** and Compound **2**. (**B**) Synthesis of the TPGSa by esterification of α-tocopherol succinate with mPEG. (**C**) ^1^H NMR spectrum of PDC in CDCl_3_. (**D**) ^1^H-NMR spectrum of the TPGSa in CDCl_3_. The asterisk (*) indicates the solvent peak. (**E**) Negatively stained TEM images of PDC nanoassemblies (scale bar = 100 nm). (**F**) Hydrodynamic diameter and zeta potential of PDC measured by DLS (n = 5). (**G**) Size distributions of PDC before and after exposure to 1 mM H_2_O_2_ at 37 °C for 3 h.

The synthesis of the PDC and TPGSa is summarized in Figure 2. The PDC was assembled through two TK motifs in series between mPEG and CPT. First, the thioketal building block DET was prepared through the acid-catalyzed condensation of acetone with 3-mercaptopropionic acid. PDT was subsequently obtained by LiAlH_4_ reduction of DET, converting the terminal carboxylic acid groups to primary hydroxyl groups. mPEG was coupled to DET under EDC/DMAP-mediated esterification conditions to afford Compound **1**, which contains one TK unit and a terminal carboxylic acid (Figure S1A). Compound **1** was then extended with PDT to generate Compound **2**, bearing two consecutive TK units and a terminal hydroxyl group (Figure S1B). Finally, the 20-OH of CPT was activated with triphosgene and coupled to Compound **2** through a carbonate linkage, yielding PDC (Figure 2A). In parallel, the TPGSa was obtained by EDC/DMAP-mediated esterification of α-tocopherol succinate with mPEG (Figure 2B).

The ^1^H-NMR spectrum of the PDC exhibited successful formation of the designed mPEG-TK-TK-CPT conjugate (Figure 2C). The thioketal methyl resonance appears at δ 1.62 ppm and is assigned to the –C(CH3)2– groups of the TK motifs. The dominant mPEG backbone resonances are observed at δ 3.55–3.84 ppm, whereas carbonate-adjacent methylene resonances appear at δ 4.2–4.4 ppm. Weak CPT aromatic resonances are detected in the δ 7.4–8.4 ppm region. A comparison of the integrated areas of CPT-derived and mPEG-derived resonances gave an estimated CPT-to-mPEG molar ratio of approximately 1:1; based on this stoichiometry, the calculated CPT content of the PDC was 12.6 wt%.

As shown in Figure 2D, the chromanol-ring methyl resonances of TPGSa appear around δ 1.8–2.1 ppm, and the phytyl-tail resonances appear in the δ 0.8–1.3 ppm region. Moreover, the succinate methylene groups are observed around δ 2.5–2.7 ppm, and the mPEG backbone is centered near δ 3.6 ppm. Together, these data support the successful synthesis of both the PDC and TPGSa used for the combination studies. The TK free LIC showed mPEG- and CPT-derived signals but no TK methyl resonance (Figure S1C), which supports formation of the intended carbonate-linked mPEG2k-CPT control.

### 3.2. Self-Assembly and Stability

Negative stained TEM showed spherical PDC nanoassemblies without large aggregates (Figure 2E). DLS gave a hydrodynamic diameter of 98.6 ± 2.6 nm and a zeta potential of −13.3 ± 1.2 mV (Figure 2F). The difference between the particle sizes measured by TEM and DLS may arise because TEM analyzes dehydrated particles, whereas DLS measures their hydrodynamic size in dispersion. The PDC displayed a narrow distribution, whereas H_2_O_2_-treated PDC showed a marked shift toward a larger and more heterogeneous size distribution (Figure 2G), consistent with the oxidative loss of amphiphilic balance and secondary aggregation of CPT-rich fragments generated during linker cleavage. The PDC remained colloidally dispersed in PBS containing 10% FBS for 4 days, with only a modest increase in hydrodynamic diameter (Figure S2A). Lyophilized PDC stored at −20 °C retained a similar reconstituted diameter and zeta potential over 28 days (Figure S2B). By contrast, the TK-free LIC did not form a well-defined nanoassembly under the same aqueous preparation conditions, exhibiting a broad and polydisperse size distribution (polydispersity index = 0.74) in DLS, with markedly inconsistent intensity-, volume-, and number-weighted size estimates. This might be due to the reduced hydrophobic content of the LIC, in which a single CPT unit is attached to the hydrophilic mPEG2k chain without the hydrophobic TK-containing segment. The LIC was therefore used only as a TK-free chemical control experiments in this study.

### 3.3. ROS-Triggered Structural Cleavage and In Vitro CPT Release

**Figure 3:**
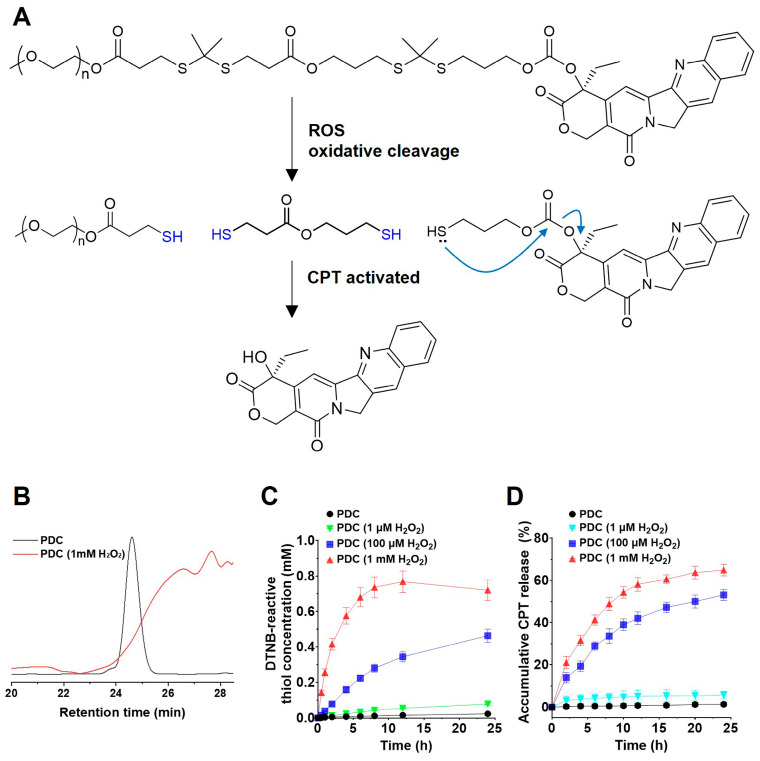
Cleavage and CPT release from the PDC. (**A**) Proposed ROS-mediated TK cleavage and putative thiol-dependent CPT-release pathway. Oxidative cleavage is expected to generate thiol-containing linker fragments, whereas the CPT-proximal thiol-carbonate species is proposed to undergo intramolecular cyclization–elimination to liberate CPT. (**B**) GPC analysis of PDC before and after H_2_O_2_ treatment. (**C**) Time-dependent generation of DTNB-reactive thiol species from PDC in the presence of H_2_O_2_. (**D**) Cumulative CPT release from PDC in PBS (pH 7.4) containing 0, 1, 100 μM or 1 mM H_2_O_2_ at 37 °C. Data are presented as mean ± SD (n = 5).

The proposed oxidative activation pathway is shown in Figure 3A. ROS-mediated cleavage of the TK motifs is expected to generate thiol-containing fragments and acetone. Cleavage near CPT is proposed to produce a thiol-containing carbonate species that may undergo intramolecular cyclization–elimination to liberate CPT. Since the intermediate structure and cyclic byproduct were not directly assigned, the scheme is therefore a working model rather than a fully validated mechanism.

To evaluate whether oxidative treatment altered the molecular size of PDC, GPC analysis was used (Figure 3B). The PDC exhibited a relatively sharp main elution peak, whereas H_2_O_2_-treated PDC showed a broadened trace, shifted toward a longer retention time, with Mn decreasing from 3190 to 1240. This shift might be due to the fragmentation of the polymer chain via cleavage of the dual-thioketal linkages. From Ellaman analysis, the PDC generated thiol species in a H_2_O_2_ concentration- and time-dependent manner, with the strongest and most rapid response under 1 mM H_2_O_2_ and a slower response at 100 μM (Figure 3C). The 1 μM and no-peroxide conditions remained near baseline over most of the assay period, supporting the oxidative transformation of the TK-containing linker in PDC.

In vitro CPT release was then evaluated in PBS containing different concentrations of H_2_O_2_ (0, 1, 100 μM, and 1 mM) at 37 °C (Figure 3D). Under non-oxidative conditions, the release of CPT from the PDC remained <2% for 24 h, indicating limited drug release. On the other hand, under 100 μM and 1 mM H_2_O_2_ conditions, 46 and 61% of CPT from the PDC was released within 24h, respectively. The TK-free LIC showed <9% CPT release with no evident H_2_O_2_ concentration-dependent behavior (Figure S2D), and its DTNB/Ellman signal remained near the assay background without a H_2_O_2_-dependent increase (Figure S2C). Therefore, this LIC comparison supports a TK linker-dependent contribution to the H_2_O_2_-responsive CPT release from PDC.

### 3.4. Cytotoxicity in A549 and BEAS-2B Cells

**Figure 4:**
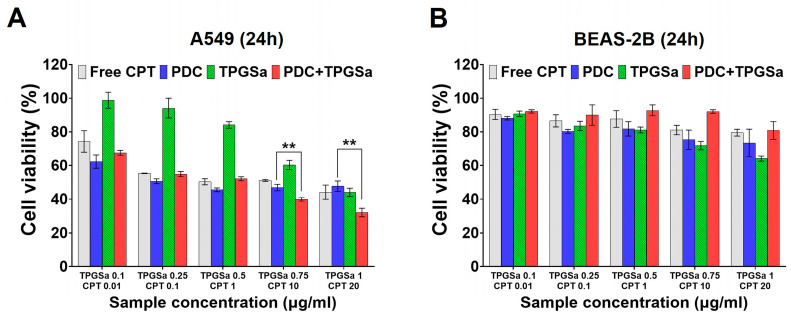
Enhanced in vitro anticancer efficacy of the PDC and TPGSa co-treatment. (**A**) Cell viability of A549 cells after 24 h treatment with free CPT, PDC, TPGSa, or PDC + TPGSa at increasing CPT-equivalent concentrations (0.01, 0.1, 1, 10, and 20 μg/mL); TPGSa was co-administered at proportional concentrations of 0.1, 0.25, 0.5, 0.75, and 1 μg/mL, respectively (*n* = 5). (**B**) Cell viability of BEAS-2B normal lung epithelial cells under the same treatment conditions (*n* = 5). Asterisks ** indicates significance level at *p* < 0.01, respectively.

To evaluate the anticancer activity of the PDC and TPGS combination, the in vitro cytotoxicity of free CPT, PDC, TPGSa, and PDC + TPGSa was assessed in A549 lung adenocarcinoma cells and BEAS-2B normal lung epithelial cells using CCK-8 viability assays (Figure 4). At 24 h, a concentration-dependent co-treatment effect emerged in A549 cells at the two upper paired-dose conditions (Figure 4A). At 10 μg/mL PDC plus 0.75 μg/mL TPGSa, PDC + TPGSa reduced A549 viability to 40.0 ± 1.1%, compared with 46.9 ± 1.9% for PDC alone and 60.3 ± 2.8% for TPGSa alone. At the highest paired-dose condition, viability decreased to 32.1 ± 2.5%, compared with 47.8 ± 3.1% for PDC alone and 44.1 ± 2.4% for TPGSa alone. Thus, PDC + TPGSa produced the strongest cytotoxicity among all treatment groups at both upper paired-dose conditions. This pattern was not observed consistently at the lower concentrations, indicating that the potentiating effect emerged primarily within the upper paired-dose range tested.

BEAS-2B cells did not reproduce the co-treatment-specific viability reduction observed in A549 cells (Figure 4B). At both upper paired conditions, PDC + TPGSa reduced BEAS-2B viability to 92.2 ± 1.0% and 76.8 ± 5.3%, respectively. These values were not lower than those obtained with the corresponding PDC or TPGSa alone. In contrast, A549 viability under the same combination conditions was 40.0 ± 1.1% and 32.1 ± 2.5%, corresponding to A549–BEAS-2B viability differences of approximately 52.2 and 44.7%, respectively. The reduction observed in A549 cells was therefore not reproduced in the non-cancerous bronchial epithelial comparison model at 24 h.

The differential response persisted after 48 h treatment (Figure S3). In A549 cells, PDC + TPGSa reduced viability to 18.0 ± 0.4% and 13.5 ± 1.1% at both upper paired conditions, respectively, compared with 29.9 ± 0.2% and 30.6 ± 0.6% for PDC alone and 43.4% and 26.1% for TPGSa alone. As expected, PDC + TPGSa exhibited the strongest anticancer activity among all groups. Although BEAS-2B viability also decreased at the upper concentrations after prolonged exposure, PDC + TPGSa did not produce an additional viability loss relative to the corresponding single treatments. Moreover, BEAS-2B viability remained substantially higher than A549 viability under the matched conditions. Collectively, these results demonstrate concentration- and time-dependent potentiation of the PDC-associated cytotoxic response in A549 cells that was not reproduced in BEAS-2B cells.

**Figure 5:**
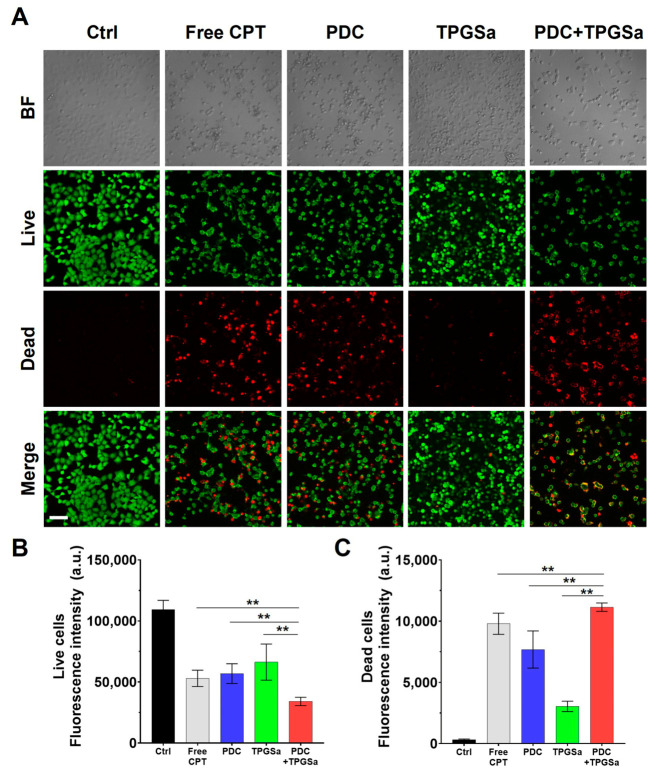
Live/dead fluorescence staining of A549 cancer cells following co-treatment with PDC and TPGSa. (**A**) Confocal fluorescence images of live/dead stained A549 cells after 15 h treatment with PBS (Ctrl), free CPT (20 μg/mL), PDC (20 μg/mL CPT-equivalent), TPGSa (1 μg/mL), or PDC + TPGSa (20 μg/mL CPT-equivalent with 1 μg/mL TPGSa). Scale bar = 100 μm. (**B**) Quantified live-cell population from the images in (**A**). (**C**) Quantified dead-cell population from the same images. Data in (**B**,**C**) are presented as mean ± SD (*n* = 5). Asterisks ** indicate significance level at *p* < 0.01.

Live/dead fluorescence staining provided morphological corroboration of the cell viability data (Figure 5A). Among the treatment groups, PDC + TPGSa exhibited the most pronounced shift from live to dead populations, with reduced green fluorescence and the highest red fluorescence intensity. Free CPT and PDC alone showed moderate levels of cell death, while TPGSa alone also induced notable cell death, consistent with its intrinsic cytotoxicity observed by CCK-8. Quantification of live and dead cells demonstrated that PDC + TPGSa showed the lowest live-cell signal and the highest dead-cell signal among all groups (Figure 5B,C). These data support TPGSa-mediated potentiation of PDC cytotoxicity in A549 cells. We next investigated whether it was accompanied by elevated intracellular ROS and increased CPT accumulation.

### 3.5. TPGSa-Amplified Intracellular ROS Generation and CPT-Associated Fluorescence from PDC in A549 Cells

**Figure 6:**
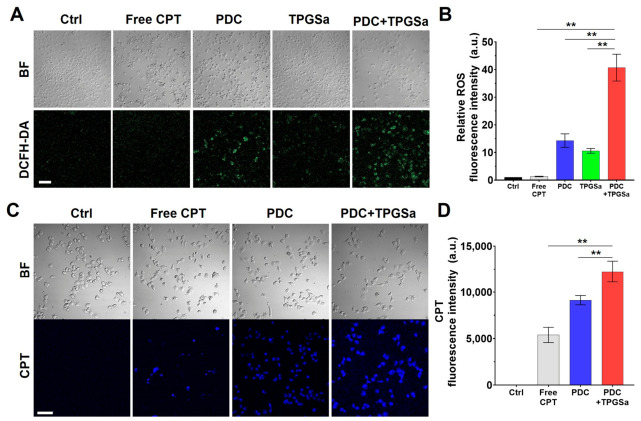
TPGS-amplified intracellular ROS generation and enhanced intracellular CPT accumulation in A549 cells. (**A**) Confocal fluorescence images of A549 cells stained with DCFH-DA after 24 h treatment with PBS (Ctrl), free CPT (20 μg/mL), PDC (20 μg/mL CPT-equivalent), TPGSa (1 μg/mL), or PDC + TPGSa (20 μg/mL CPT-equivalent with 1 μg/mL TPGSa). DCFH-DA (green, λ_ex_/λ_em_ = 488/525 nm). BF = bright field. Scale bar = 100 μm. (**B**) Quantified ROS fluorescence intensity of DCFH-DA in A549 cells. Data are presented as mean ± SD (*n* = 5). (**C**) Confocal fluorescence images of A549 cells after 4 h treatment with PBS (Ctrl), free CPT (20 μg/mL), PDC (20 μg/mL CPT-equivalent), or PDC + TPGSa (20 μg/mL CPT-equivalent + 1 μg/mL TPGSa). CPT (blue, λ_ex_/λ_em_ = 352/402 nm). BF = bright field. Scale bar = 100 μm. (**D**) Quantified CPT fluorescence intensity of A549 cells. Data are presented as mean ± SD (*n* = 5). Asterisks ** indicate significance level at *p* < 0.01.

To assess whether the enhanced cellular activity was associated with intracellular ROS elevation, intracellular oxidant levels were evaluated in A549 cells using the cell-permeant DCFH-DA probe, which is oxidized by intracellular oxidants to highly fluorescent DCF (Figure 6A,B). Notably, PDC + TPGSa showed the highest ROS-associated fluorescence signal among the tested groups. Free CPT did not raise the DCFH-DA signal above the control. By contrast, TPGSa alone increased the signal relative to the control, and PDC alone also produced a measurable increase. This might be attributable to the PDC-associated redox response, including its nanoparticle structure and the oxidative cleavage of the TK linkers.

To examine whether the elevated intracellular ROS environment was accompanied by altered intracellular CPT accumulation, the intrinsic fluorescence of CPT was imaged (Figure 6C). CPT is intrinsically fluorescent, which allows its intracellular distribution to be visualized directly without an additional label, an approach widely used for CPT- and camptothecin-derivative-based delivery systems [29]. PDC + TPGSa produced the highest CPT-associated intracellular fluorescence among the tested groups, exceeding that of PDC alone and free CPT. Quantitatively, the CPT-associated signal in A549 cells was 2.3- and 1.4-fold higher for PDC + TPGSa than for free CPT and PDC alone, respectively (Figure 6D). Although the intrinsic fluorescence of CPT cannot distinguish free CPT from intact PDC or from CPT-containing degradation species, the higher CPT-associated signal in the PDC + TPGSa group is consistent with the enhanced cytotoxicity observed in A549 cells. These results, together with in vitro release and Ellman data, support that TPGSa-associated ROS elevation is consistent with an increased intracellular CPT-associated signal.

## 4. Discussion

The present study defines PDC and TPGSa as functionally connected components of an in vitro ROS-modulated CPT delivery strategy. PDC self-assembled into PEG-shielded nanoassemblies with a hydrodynamic diameter of approximately 100 nm. In PBS containing 10% FBS, the PDC nanoassemblies showed good colloidal stability, indicating resistance to serum-induced aggregation. Although long-term stability under in vivo conditions remains to be established, the lyophilized PDC also retained its diameter and zeta potential during storage at −20 °C, supporting practical handling in vitro. Under peroxide conditions, the PDC showed molecular size changes, thiol generation, and H_2_O_2_ concentration-dependent CPT release. In particular, in A549 cells, PDC + TPGSa produced the strongest DCFH-DA signal, CPT-associated fluorescence, dead-cell staining, and upper-dose viability reduction. Taken together, these findings support a working model in which TPGSa-associated redox perturbation can accompany enhanced activity of the ROS-responsive PDC.

The dual-TK design localizes two ROS-cleavable motifs at the polymer–drug junction and differs from both conventional single-TK conjugates and poly(thioketal) backbones [14,16,17,19]. It is presented as a proof-of-concept linker-level architecture rather than as a demonstration of superiority over single-TK PDCs because the present study did not include a physicochemically matched single-TK comparator. Instead, the TK-free LIC addressed a distinct mechanistic question: whether the peroxide response depended on ROS-cleavable TK chemistry. Because the LIC did not form a well-defined nanoassembly comparable to the PDC, it was used as a TK-free chemical control rather than as a formulation-matched comparator. It showed only background-level DTNB responses and low CPT release with no meaningful H_2_O_2_ concentration-dependent increase, supporting a TK linker-dependent contribution to the peroxide responsiveness of the PDC. A physicochemically matched single-TK or linker length-matched comparator will be required to isolate the contribution of linker multiplicity, which remains necessary future work.

The combined GPC, Ellman test, and HPLC-based release results provide converging chemical evidence for oxidative activation of the PDC. The concentration-dependent thiol response, together with the peroxide-dependent size change observed in GPC and the H_2_O_2_-dependent CPT release, is consistent with oxidative processing of the TK-containing linker. These results strengthen the interpretation that TK cleavage initiates CPT liberation. While these methods collectively support the cleavage process, full structural assignment of the CPT-side intermediate and the cyclic byproduct will require more definitive analytical approaches, such as LC-MS/MS, for further studies.

TPGSa was used as a soluble, separately administered PEG2000-based agent rather than as an independent micellar carrier. Conventional TPGS-1000 and tocopheryl succinate-derived systems have been associated with mitochondrial perturbation and ROS-linked cytotoxicity [23,24,25,30], including a carrier-integrated F127-SS-TPGS micelle that released TPGS and increased intracellular oxidative stress. The current DCFH-DA data are consistent with TPGSa-associated redox perturbation, but they do not establish a mitochondrial complex II mechanism or direct ROS causality.

The comparison between A549 and BEAS-2B cells showed that, at both 24 and 48 h, PDC + TPGSa produced the lowest A549 viability among all treatment groups at the two upper paired-dose conditions. The differences between the combination and the corresponding single treatments became greater with prolonged exposure. In BEAS-2B cells, however, PDC + TPGSa did not reduce viability below that observed with the corresponding single treatments, suggesting that the combination-specific response observed in A549 cells was not reproduced in the non-cancerous bronchial epithelial comparison model. Nevertheless, TPGSa alone became increasingly cytotoxic at the upper concentrations, and an additive contribution to the overall co-treatment response cannot be excluded. Because a full fixed-ratio dose matrix and formal combination analysis were not performed, the observed effect is described as potentiation rather than pharmacological synergy. Free CPT also showed cell line-dependent cytotoxicity, indicating that intrinsic CPT sensitivity, proliferation rate, topoisomerase-I dependence, DNA-damage responses, cellular uptake or retention, and antioxidant buffering may contribute to the A549/BEAS-2B difference. Overall, these findings support a dose- and time-dependent in vitro differential sensitivity profile rather than definitive selective PDC activation. Accordingly, the highest tested paired-dose condition should be regarded as the maximal-response condition within the evaluated range, not as an optimized or safe dose.

Finally, the present work is limited to in vitro physicochemical and cellular evaluation. Pharmacokinetics, biodistribution, efficacy, and systemic safety will require a separately designed and adequately powered in vivo study. Within these boundaries, the study provides a strengthened proof-of-concept framework for combining an ROS-responsive PDC with a soluble TPGSa while explicitly separating evidence for TK-dependent peroxide responsiveness from unresolved questions of linker multiplicity, intracellular mechanisms, and translational performance. This approach may help guide the design of ROS-responsive polymeric prodrugs for cancer cell microenvironments in which basal oxidative stress may be insufficient for efficient drug release.

## 5. Conclusions

A dual-TK mPEG2k–CPT conjugate was synthesized and shown to form approximately 100 nm PEG-shielded nanoassemblies with good colloidal stability in a protein-containing medium and after lyophilized storage. The PDC exhibited peroxide concentration-dependent structural changes, thiol generation, and CPT release, whereas the TK-free LIC showed no meaningful H_2_O_2_-dependent increase in either thiol generation or CPT release. Co-treatment with TPGSa increased ROS-associated and CPT-associated fluorescence and potentiated PDC cytotoxicity in A549 cells at the upper paired-dose conditions. BEAS-2B cells showed a weaker response at 24 h, although toxicity increased in both cell lines with longer exposure. Together, these findings support a dose-, time-, and cell type-dependent in vitro differential response rather than synergy or selective PDC activation. Overall, the study provides an in vitro proof of concept that TPGSa-associated redox perturbation can accompany enhanced activity of an ROS-responsive CPT-PDC under conditions where basal oxidative stress alone may be insufficient for efficient drug release.

## Data Availability

The raw data supporting the conclusions of this article will be made available by the authors on request.

## Supplementary Materials

The following supporting information can be downloaded at: https://www.mdpi.com/article/10.3390/pharmaceutics18070886/s1, Figure S1: ^1^H NMR spectra of mPEG2k–TK (Compound **1**), mPEG2k–TK-TK (Compound **2**) and LIC. Figure S2: Time-dependent hydrodynamic diameter of PDC in the presence of PBS containing 10% FBS for 4 days. (B) Storage stability of lyophilized PDC at −20 °C over 28 days. (C) DTNB/Ellman analysis of LIC in the presence or absence of H_2_O_2_ for 24 h. (D) Cumulative CPT release from LIC in the presence or absence of H_2_O_2_ for 24 h. Figure S3: Enhanced in vitro anticancer efficacy of the PDC and TPGSa co-treatment. (A) Cell viability of A549 cells after 48 h treatment with free CPT, PDC, TPGSa, or PDC + TPGSa at increasing CPT-equivalent concentrations. (B) Cell viability of BEAS-2B normal lung epithelial cells under the same treatment conditions.

## Abbreviations

The following abbreviations are used in this manuscript:

ROS	Reactive oxygen species
PDC	Polymeric drug conjugate
TK	Thioketal
TPGSa	mPEG2k–tocopheryl succinate (mPEG2k–TS)
CPT	Camptothecin
mPEG	Methoxy poly(ethylene glycol)
DLS LIC	Dynamic light scattering Linker-insensitive control
TEM	Transmission electron microscopy
GPC	Gel permeation chromatography
NMR	Nuclear magnetic resonance
DCFH-DA	2′,7′-Dichlorofluorescein diacetate
CCK-8	Cell Counting Kit-8
PBS	Phosphate-buffered saline
FBS	Fetal bovine serum
DET	Diethyl thioketal
PDT	Propanediol thioketal
HPLC	High-performance liquid chromatography
EDC	1-Ethyl-3-(3-dimethylaminopropyl)carbodiimide
DMAP	4-Dimethylaminopyridine
SD	Standard deviation
