# Supplementary material for: TPGS Analog-Mediated Intracellular ROS-Amplifying Strategy Potentiates the In Vitro Anticancer Activity of a Dual-Thioketal-Linked Polymeric Drug Conjugate in A549 Lung Cancer Cells
Source: Pharmaceutics. 2026 Jul 20;18(7):886. doi: 10.3390/pharmaceutics18070886 (PMC13416317; doi:10.3390/pharmaceutics18070886)
Supplement: Supplementary file 1 [file pharmaceutics-18-00886-s001.zip › pharmaceutics-4384034-supplementary.pdf]

Article

Supplementary Materials: TPGS Analog-Mediated Intracellular ROS-Amplifying Strategy Potentiates the In Vitro Anticancer Activity of a Dual-Thioketal-Linked Polymeric Drug Conjugate in A549 Lung Cancer Cells

Hyun-Chul Kim[1,†], Kyeong-Min Lee[1,†], Yeo Jin Hwang[2], ,Jonghun Lee[3,*], Hwa Seung Han[1,†,*]

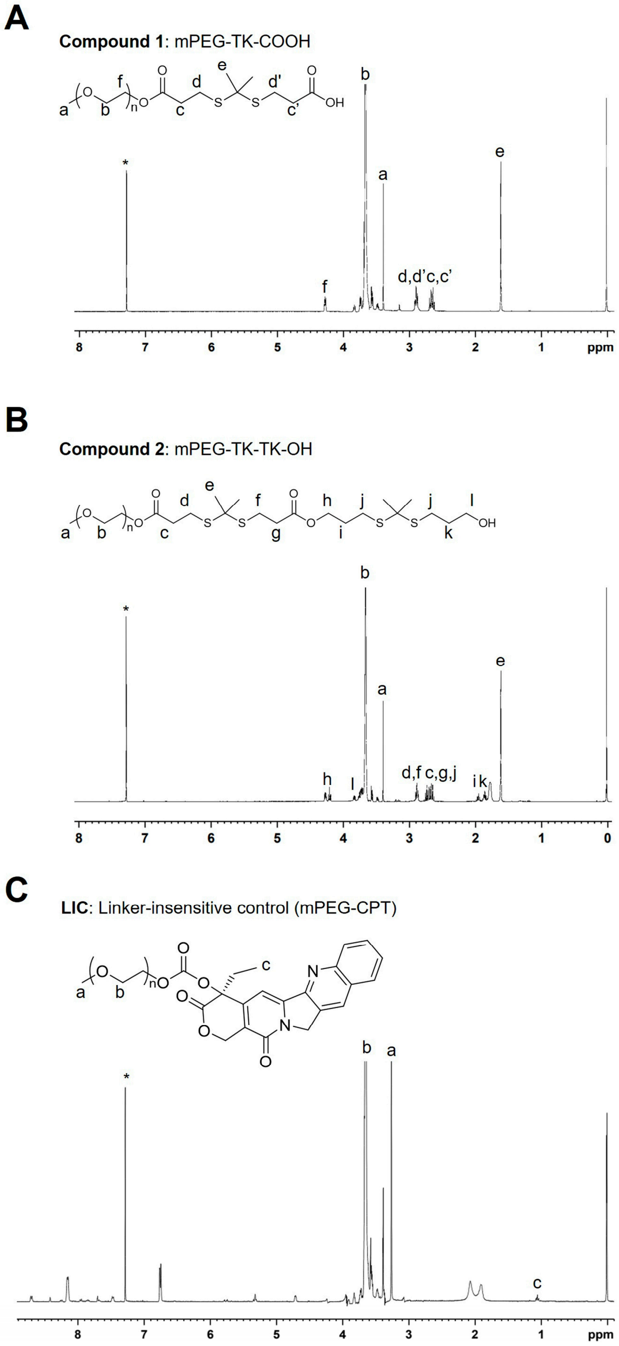

Figure S1. ^{1}H-NMR spectrum of the Compound 1(A), Compound 2 (B) and LIC (C) in $CDCl_3$.

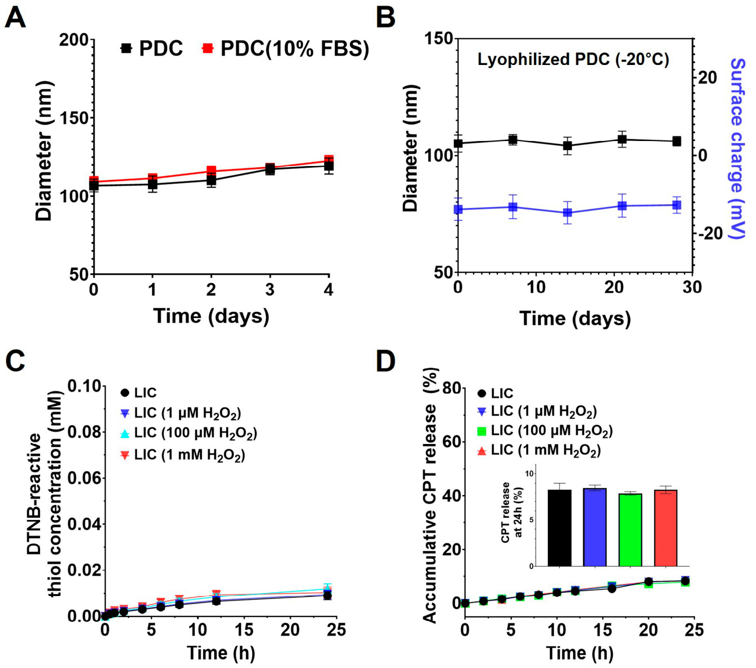

Figure S2. (A) Time-dependent hydrodynamic diameter of PDC in the presence of PBS containing 10% FBS for 4 days. (B) Storage stability of lyophilized PDC at -20 °C over 28 days, assessed by hydrodynamic diameter and zeta potential after reconstitution. (C) DTNB/Ellman analysis of LIC in the presence or absence of H_2O_2 for 24 h. (D) Cumulative CPT release from LIC in the presence or absence of H_2O_2 for 24 h; the inset summarizes cumulative release at 24 h. Data are presented as mean +/- SD (n = 5)

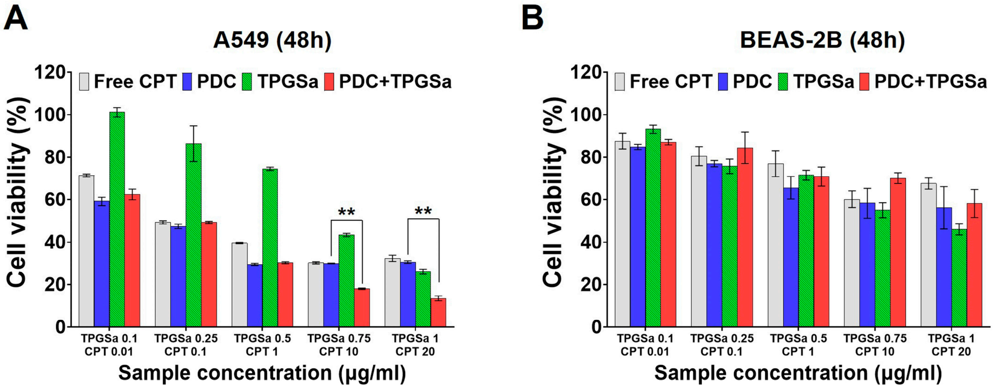

Figure S3. Enhanced *in vitro* anticancer efficacy of the PDC and TPGSa co-treatment. **(A)** Cell viability of A549 cells after 48 h treatment with free CPT, PDC, TPGSa, or PDC+TPGSa at increasing CPT-equivalent concentrations (0.01, 0.1, 1, 10, and 20 μg/mL); TPGS was co-administered at proportional concentrations of 0.1, 0.25, 0.5, 0.75, and 1 μg/mL, respectively (n = 5). **(B)** Cell viability of BEAS-2B normal lung epithelial cells under the same treatment conditions (n = 5). Asterisks * and ** indicate significance level at $p < 0.05$ and $p < 0.01$, respectively.
